# When Outgroups Fail; Phylogenomics of Rooting the Emerging Pathogen, *Coxiella burnetii*

**DOI:** 10.1093/sysbio/syt038

**Published:** 2013-07-22

**Authors:** Talima Pearson, Heidie M. Hornstra, Jason W. Sahl, Sarah Schaack, James M. Schupp, Stephen M. Beckstrom-Sternberg, Matthew W. O'Neill, Rachael A. Priestley, Mia D. Champion, James S. Beckstrom-Sternberg, Gilbert J. Kersh, James E. Samuel, Robert F. Massung, Paul Keim

**Affiliations:** ^1^Center for Microbial Genetics and Genomics, Northern Arizona University, Flagstaff, AZ, USA; ^2^Department of Biology, Reed College, Portland, OR, USA; ^3^Pathogen Genomics Division, Translational Genomics Research Institute, Flagstaff, AZ, USA; ^4^Rickettsial Zoonoses Branch, Centers for Disease Control and Prevention, Atlanta, GA, USA; ^5^Department of Microbial and Molecular Pathogenesis, Texas A&M Health Science Center, College Station, TX, USA

## Abstract

Rooting phylogenies is critical for understanding evolution, yet the importance, intricacies and difficulties of rooting are often overlooked. For rooting, polymorphic characters among the group of interest (ingroup) must be compared to those of a relative (outgroup) that diverged before the last common ancestor (LCA) of the ingroup. Problems arise if an outgroup does not exist, is unknown, or is so distant that few characters are shared, in which case duplicated genes originating before the LCA can be used as proxy outgroups to root diverse phylogenies. Here, we describe a genome-wide expansion of this technique that can be used to solve problems at the other end of the evolutionary scale: where ingroup individuals are all very closely related to each other, but the next closest relative is very distant. We used shared orthologous single nucleotide polymorphisms (SNPs) from 10 whole genome sequences of *Coxiella burnetii*, the causative agent of Q fever in humans, to create a robust, but unrooted phylogeny. To maximize the number of characters informative about the rooting, we searched entire genomes for polymorphic duplicated regions where orthologs of each paralog could be identified so that the paralogs could be used to root the tree. Recent radiations, such as those of emerging pathogens, often pose rooting challenges due to a lack of ingroup variation and large genomic differences with known outgroups. Using a phylogenomic approach, we created a robust, rooted phylogeny for *C. burnetii*. [*Coxiella burnetii*; paralog SNPs; pathogen evolution; phylogeny; recent radiation; root; rooting using duplicated genes.]

Rooting is an important aspect for phylogenetic inference and understanding evolutionary change. When properly rooted, the character states of the hypothetical common ancestor can be inferred, the directionality of evolution for each character can be deduced, and the relative rate of evolution can be estimated (Throckmorton 1968; Farris 2010). Also, a rooted tree can inform the relationships among taxa and clades (Huelsenbeck et al. 2002) and be used to determine geographic dispersal and adaptations, critical elements to epidemiological and forensic investigations of bacterial pathogens (Kenefic et al. 2009; Morelli et al. 2010; Pearson et al. 2009; Hendriksen et al. 2011).

Outgroup, molecular clock, and midpoint rooting approaches are commonly used to infer the position of the root. Unfortunately, for molecular clock and midpoint rooting, the requisite underlying assumption of equal evolutionary rates is rarely met (Swofford et al. 1996). For successful outgroup rooting, polymorphic sites among ingroup taxa must be found in the outgroup. A distant outgroup can increase the likelihood of homoplasy (convergent evolution resulting in character state conflicts in a given tree) which, in the absence of closer outgroups and coupled with unequal and short branch lengths, could result in an incorrect root assignment (Wheeler 1990; Huelsenbeck et al. 2002). Thus, two characteristics have a direct bearing on rooting: diversity within the ingroup and evolutionary distance to the outgroup. Rapid and recent radiations can be particularly difficult to root as they are often characterized by low ingroup diversity and relatively long phylogenetic distances to their nearest neighbor taxa (Shavit et al. 2007). Such rapid radiations are characteristic of many newly emerged pathogens and *Coxiella burnetii*, the causative agent of Q fever in humans, provides a good example of this challenge.

*Coxiella burnetii* is an intracellular pathogen that infects a wide range of vertebrates, especially livestock, and occasionally humans (Maurin and Raoult 1999). In most human cases, Q fever is a self-limiting disease with flu-like symptoms, but occasionally it develops into a chronic infection and can lead to endocarditis and death (Maurin and Raoult 1999). *Coxiella burnetii* has the potential for rapid dispersal because, during parturition, infected animals shed large numbers of bacteria that are easily aerosolized and highly infectious, with *<*10 organisms needed to cause disease via inhalation (Benenson and Tigertt 1956).

The evolutionary origins of *C. burnetii* remain a mystery as outgroup species cannot be used to root this group. It is the sole known representative of its genus and the closest known neighbors (*Legionella* spp. and *Ricketsiella grylli*) are evolutionarily distant (Roux et al. 1997). A lack of genomic diversity among *C. burnetii* isolates (Beare et al. 2006; Beare et al. 2009) also complicates rooting. Genomic rearrangements, thought to be mediated by insertion sequences (IS), account for most variation (Beare et al. 2009), with a limited number of indels (Beare et al. 2006) and rare SNPs (Glazunova et al. 2005) also contributing. Here, we show that whole genome comparisons reveal many evolutionarily stable single nucleotide polymorphisms (SNPs) that can be used to create a robust phylogeny of *C. burnetii*. Because few of these sites are shared with potential outgroups, the phylogenetic location of the root has previously been unknown. This, in turn, has impeded our understanding of the evolutionary relationships among existing lineages and made it impossible to definitively determine the origins of current outbreaks.

Rapid radiations, such as that of *C. burnetii*, do not represent the only rooting challenge in phylogenetics. For example, how can rooting be accomplished if there is no outgroup, such as for the tree of life (Gogarten et al. 1989; Iwabe et al. 1989)? Rooting is also difficult when there is significant divergence from the nearest relatives (Telford and Holland 1997; Hendrix et al. 1999; Mathews and Donoghue 1999; Simmons 2008; Brady et al. 2011; Ness et al. 2011), or when outgroup taxa are not firmly established (Outlaw and Ricklefs 2011). One approach has been the use of duplicated or paralogous genes where the duplication events are older than the phylogenetic group being analysed (Gogarten et al. 1989; Iwabe et al. 1989; Telford and Holland 1997; Mathews and Donoghue 1999; Brady et al. 2011). The presence of highly similar genomic regions in different genomic locations in all ingroup taxa is indicative of a duplication event that occurred prior to the last common ancestor (LCA) (Fig. 1). The subsequent divergence of such paralogs prior to the LCA is akin to the divergence of an outgroup, and thus can be treated as such for rooting purposes.

**Figure 1:**
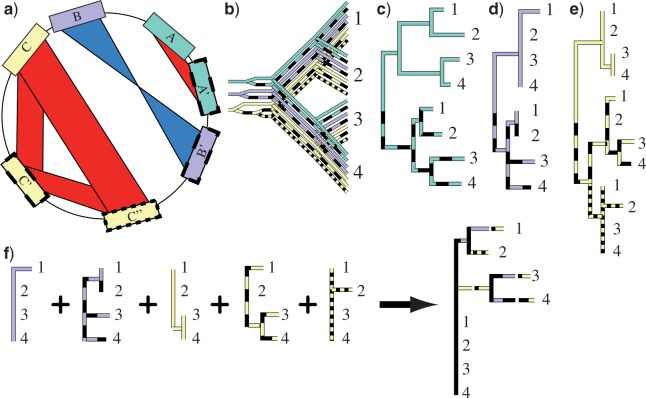
Schematic of paralogous evolution and rooting. a) Duplicated regions on bacterial chromosomes can be identified by high levels of similarity, including inverted (regions B and B^′^) or sequential duplications (C, C^′^, and C^″^). b) The evolutionary history of the three genomic regions from (a) among four taxa (1–4) shows four duplication events occurring at different times but, critically for rooting, before speciation events. c–e) Phylogenetic trees reflecting paralog evolution can be expected to contain a clade specific for each paralog. c) For diverse groups, a single duplicated region may contain enough polymorphisms to fully resolve the tree. The point at which the paralog clades connect will identify the phylogenetic duplication point and, for a sufficiently resolved tree, indicate that the root lies between the clades 1 and 2 and 3 and 4. d, e) For groups with little diversity, single paralog sets may not resolve the tree sufficiently for rooting, especially as the phylogenetic resolution is divided between paralog clades. f) A genome-wide approach that combines phylogenetic information across paralogs and duplicated regions [e.g. (d, e)] into a single clade will increase phylogenetic resolution. Paralogs are shown with solid, dashed, or dotted lines with a different color for each paralog set.

For paralogs to be useful for rooting, orthologs of each paralog must be identifiable in the other taxa and orthologs of at least one paralog must be polymorphic among other taxa. In previous work using duplicated genes for rooting, each taxon is listed multiple times; once for each paralog (Fig. 1c–e). The position where the clades of each paralog meet identifies the phylogenetic duplication point and indicates the root. For groups with little diversity, this rooting method can be modified, as single paralogs are likely to contain too few polymorphisms, leaving insufficient resolution to discern the root position (Fig. 1d,e).

Combining the phylogenetic data from across paralogous regions can increase resolution and the likelihood of identifying the root position. Using regions from across the genome also reduces the likelihood of biases due to past horizontal gene transfer events. Here, we maximize phylogenetic resolution by combining data from different paralogous regions as well as across paralog clades (Fig. 1f). Thus, each SNP, rather than a region or gene, acts as the paralog unit. With this whole genome paralog SNP approach, phylogenetic resolution can be concentrated into a single clade, rather than being divided among paralogous clades containing the same taxa. With all resolution relegated to one clade, the monomorphic paralogous clade can serve as an outgroup (Fig. 1f) in the same way that entire paralog regions have been used as outgroups to each other.

We demonstrate this whole genome paralog SNP rooting approach to root a more extensive and robust (but unrooted) phylogeny of *C. burnetii*. The unrooted phylogeny was created using shared orthologous SNPs from whole genome sequences of 10 diverse *C. burnetii* isolates. For recently emerged and clonally reproducing taxa, SNPs are ideal for constructing well resolved and well-supported phylogenies. The low mutation rate for SNPs (Lenski et al. 2003; Keim et al. 2004; Vogler et al. 2007) and the short evolutionary time frame minimizes the chance of the same nucleotide mutating again among either descendants or in independent lineages. Thus, once an SNP arises, all descendants will inherit the novel character state which will not exist in other lineages. A single SNP can therefore define a lineage and, when combined with other SNPs that all give a consistent phylogenetic signal, will result in a well-resolved, well-supported, and possibly highly accurate phylogeny (Pearson et al. 2004; Li et al. 2008; Pearson et al. 2009). We established the root for this phylogeny by drawing and rooting an independent tree of six assembled (or partially assembled) *C. burnetii* genomes using the whole genome paralogous SNP approach described above. We then placed the root in a corresponding position on the larger, better supported tree created with orthologous SNPs.

## METHODS

### Unrooted Phylogeny of C. burnetii

**Figure 2:**
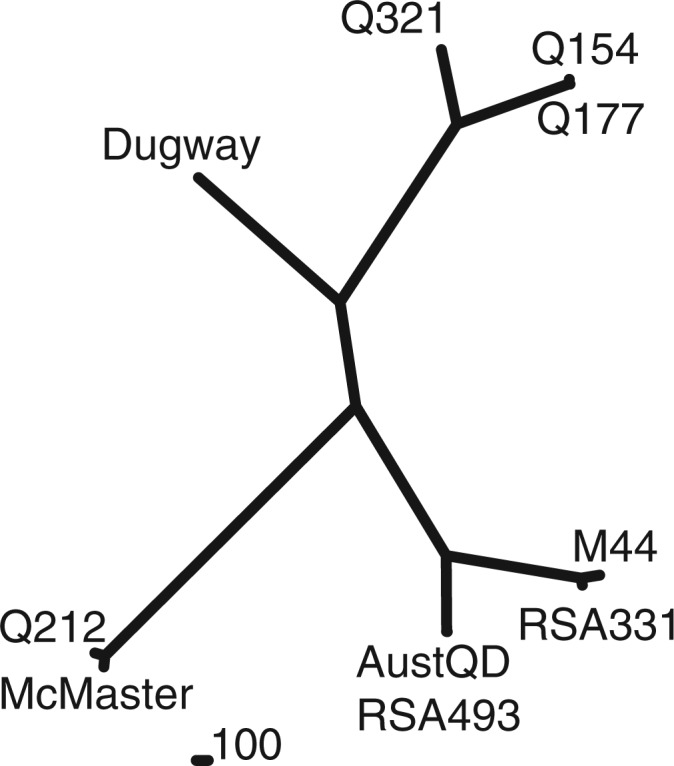
Unrooted *C. burnetii* phylogenetic tree. Unrooted maximum parsimony tree drawn with 11,386 orthologous-shared SNPs from whole genome comparisons. Only 91 SNPs were homoplastic, leading to a high consistency index of 0.9894 and 100% bootstrap support for each bifurcation.

We used MUMmer (Kurtz et al. 2004) to search for homologous genomic regions across five assembled, one partially assembled, and four short-read *C. burnetii* genomes (Table 1). Homologous regions were aligned and searched for SNPs using SolSNP (http://sourceforge.net/projects/solsnp/ last accessed June 19, 2013). We ensured site orthology by eliminating potential paralogs and regions that mapped to more than one genomic location on an assembled reference genome, as well as requiring alignment of other genomes to 100 bp flanking each side of the SNP. We minimized potential alignment errors by eliminating SNPs immediately adjacent to other polymorphisms. By requiring that all taxa share each site, we did not have to make any assumptions regarding likely character states before a site was lost. Such filtering left us with 11,386 SNP sites. SNPs were in pseudogenes (*n* = 1924), intergenic regions (*n* = 1288), ribosomal genes (*n* = 2), tRNA genes (*n* = 8) were non-synonymous (*n* = 5107), and synonymous (*n* = 3057). We used parsimony criteria and a heuristic search with default options using PAUP (Swofford 2003) to recover a single most parsimonious tree (Fig. 2). We performed 1000 bootstrap iterations to test the robustness of the tree, however as bootstrapping is a poor method for measuring accuracy for trees with little homoplasy (Felsenstein 1985), we also report homoplasies as a more appropriate and direct measure of accuracy (Archie 1996).

**Table 1.** Genomes used in this study

Species	Strain	Alternate names	GenBank ID
*Coxiella burnetii*	Q321		AAYJ00000000
	Q154	K Q154	CP001020.1
	Q177	MSU Goat Q177	AAUP00000000
	Dugway	Dugway 5J108-111	CP000733.1
	Q212	G Q212	CP001019.1
	McMaster	McMaster Placenta	SRX082468
	AustQD	Australia QD	SRX082467
	RSA493	Nine Mile Phase I	AE016828.2
	M44		SRX119301
	RSA331	Henzerling	CP000890.1
*Legionella pneumophila*	Paris		CR628336.1
	Lens		CR628337.1
*Ricketsiella grylli*			AAQJ02000001.1
*Pseudomonas syringae*	B728a		NC_007005.1

### Assessing Outgroup Taxa

As trees are traditionally rooted using outgroup taxa, we initially attempted to root the *C. burnetii* tree with other species (Table 1). Using the SNP discovery pipeline described above, we included potential outgroup genomes. In order to increase the likelihood of identifying appropriate sites, we dropped the requirement that sites are shared among all *C. burnetii* genomes because some have deleted regions and may have regions that lacked sequencing coverage. *Legionella pneumophila* and *R. grylli* are reportedly the nearest neighbors to *C. burnetii* (Roux et al. 1997), however as comparisons to *C. burnetii* yielded few appropriate sites for rooting, we also included *Pseudomonas syringae* because of high levels of sequence similarity at the 16S locus identified when using *C. burnetti* as a query to search GenBank (see the Supplementary Material S2).

### Finding, Assessing, and Rooting with Duplicated Regions

**Figure 3:**
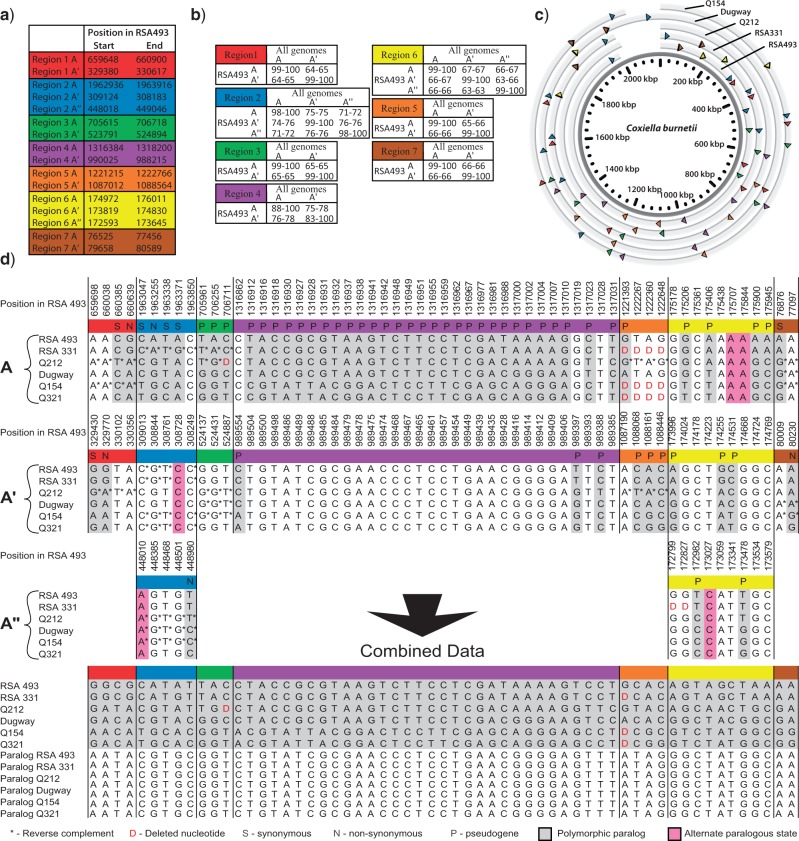
Duplicated regions used for rooting the *C. burnetii* phylogeny. a) Genomic position of paralogous regions in RSA493. Paralogs are denoted as A, A^′^, and A^″^. b) Minimum and maximum percent match between orthologs and paralogs of six genomes compared to RSA493. High values across orthologous regions and low values across paralogs ensure that orthologs for each region can be identified and distinguished from paralogs. c) Chromosomal map showing paralogs and orthologs of each region for the five assembled genomes. d) SNP states for orthologs and paralogs across six genomes. The polymorphic paralogs for each SNP site were combined into a single data set to concentrate all phylogenetic resolution into a single clade. Likewise, the monomorphic paralogs for each locus were combined so serve as an outgroup to root the resulting tree.

**Figure 4:**
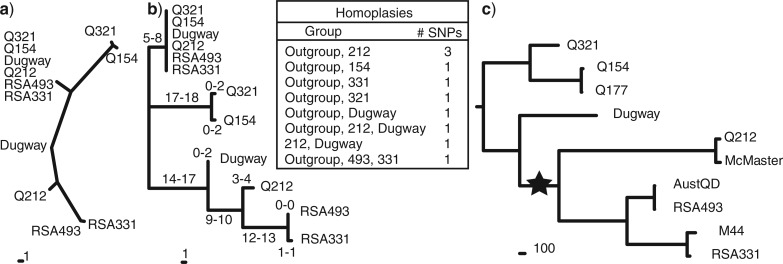
Rooting the *C. burnetii* phylogeny. a) Unrooted maximum parsimony tree using 59 SNPs from duplicated genomic regions and showing two paralogous clades. The position of the root is the point where the two paralog clades meet. All characters are parsimony informative among ingroup taxa and the consistency index is 0.8551. The minimum and maximum number of SNPs on each branch is shown along with a description of the phylogenetic groupings for the 10 homoplastic SNPs (inset). The presence of branches leading to the outgroup and to all other termini is due to homoplasy. b) Maximum parsimony tree from (a) rooted with the paralog clade as an outgroup proxy. c) The position of the root as determined in (b) is used to root the whole genome SNP tree from Figure 2. Other *C. burnetii* phylogenies have been rooted using the tree mid-point (Glazunova et al. 2005), but a midpoint root for this tree (star) is not supported by our results.

We found duplicated regions by searching for sequence similarities within the RSA493 genome (megablast of the RSA493 genome against itself). We discarded regions within 1 kb of insertion (IS) elements and transposases to minimize phylogenetic error as these regions are not likely to be evolutionarily stable. One of the resulting 12 duplicated regions longer than 900 bp was discarded as it was not a discrete duplication because it overlapped with itself and was part of another duplicated region. Sequences similar to the remaining 11 duplicated regions were identified in each of the five assembled and one partially assembled *C. burnetii* genomes using the BLASTN algorithm to determine their presence/absence and identify orthologs of each paralog. Four of the 11 duplicated regions were then eliminated from further analyses. The first eliminated region contained polymorphisms that were all parsimony uninformative and thus uninformative for rooting. In the second, multiple degenerative paralogs were identified, causing concerns of correctly identifying orthologs of each paralog in other genomes. The third region was part of another paralog, and a fourth region was discarded because it was not duplicated in other genomes and had a complex VNTR-like region, also causing concern for correctly identifying orthologs. Of the seven remaining duplicated regions, an additional paralog was identified for two of the regions, resulting in five duplicated and two triplicated regions (Fig. 3). Orthologs were identified by the best match and confirmed by comparing the flanking sequence around the region across the surveyed genomes. Alignments of orthologs and paralogs using ClustalW in BioEdit (http://www.mbio.ncsu.edu/bioedit/bioedit.html last accessed June 19, 2013) were used to find SNP polymorphisms among orthologs for each genome and identify the paralogous SNP site. Polymorphic paralogs for each SNP site were combined and the monomorphic paralogs assigned to a duplicated set of taxa that was used as an outgroup to root the resulting tree (Fig. 4). In our dataset, at least one of the polymorphic character states matched the state of a paralogous SNP. This was not a requirement, but avoids employing assumptions about the likelihood of different nucleotide changes. We used parsimony criteria as described above to recover a single most parsimonious tree. We also performed a Bayesian analysis using MrBayes (Ronquist and Huelsenbeck 2003); as only variable sites are included in the data matrix (Fig. 3d), we converted the nucleotide data into standard characters and employed the Mkv model using equal rates and state frequencies. The resulting tree showed an identical topology to the maximum parsimony tree with clade credibility values of 100% at all nodes.

### Analysis of Gene Differences

**Figure 5:**
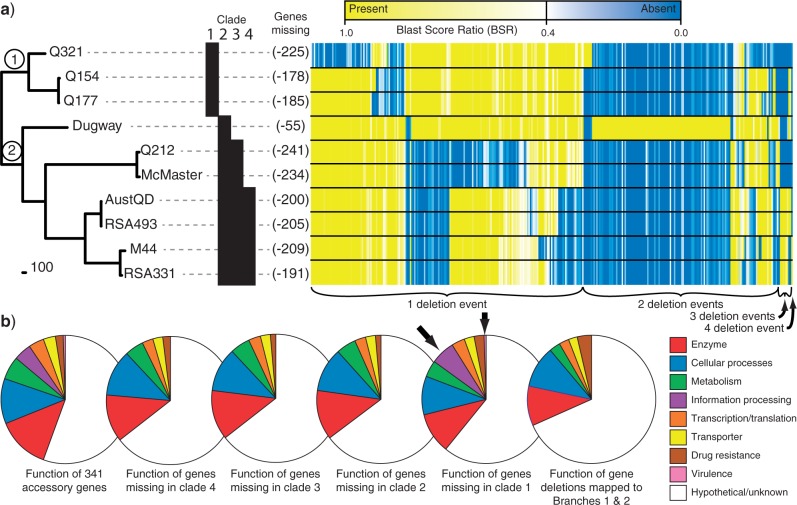
Genome reduction in *C. burnetii*. a) Rooted phylogeny of *C. burnetii* showing clade designations for reference in part (b), the number of genes deleted from each taxon and a heatmap showing the BSR for each of the 341 genes differentially present among genomes. Each gene is represented by a thin vertical line, the color of which represents its presence or absence according to a BSR cutoff of 0.4. b) Functional classification of these accessory genes missing in different phylogenetic groups. Although the functional profiles of missing gene content were similar across phylogenetic groups, genes associated with information processing and virulence were only missing from members of clade 1.

Open reading frames (ORFs) were predicted in each genome using glimmer3 (Delcher et al. 2007). ORFs were de-replicated by clustering with USEARCH (Edgar 2010) at an ID of 0.9; ORFs were then translated with Transeq (Rice et al. 2000). Each peptide was aligned to each genome with TBLASTN (Altschul et al. 1997) and the bit score value was tabulated. The conservation of peptides in each genome was determined with a blast score ratio (BSR) analysis (Rasko et al. 2005; Sahl et al. 2013); the BSR value can range from 1.0 (exact peptide match) to 0.0 (no significant alignment). Lost genes were identified as those with a BSR value of ≤0.4, equivalent to ≤40% peptide identity over 100% of the peptide length. The functional annotation of selected peptides was performed by the Kegg automatic annotation server (http://www.genome.jp/tools/kaas/ last accessed June 19, 2013). For those peptides that could not be assigned to a Kegg orthology group, an alignment to the Genbank non-redundant (nr) database was performed; the annotation was then transferred from annotated genes to peptides identified in this study. Some genes (*n* = 32) were contaminants from host cells used for culturing (rabbit and chicken) or another bacterium known to be sequenced at the same time as M44 and thus excluded from further investigations. Genes deleted from the first two phylogenetic branches (Fig. 5a) were identified by mapping possible deletions as close to the root as possible.

## RESULTS

### Unrooted Phylogeny of C. burnetii

Using 11,386 SNPs (Supplementary Material S1), of which 9096 were parsimony informative, we recovered a single maximum parsimony tree on which only 91 characters are inferred to be homoplastic. The consistency index (excluding parsimony uninformative characters) was therefore 0.9894 (Fig. 2). Relationships shown are congruent with other phylogenetic analyses of *C. burnetii* (Glazunova et al. 2005; Beare et al. 2006; Beare et al. 2009; Hornstra et al. 2011). The high consistency index suggests a completely clonal mode of genetic inheritance, with little, if any, evidence for lateral gene transfer among *C. burnetii* genomes. The high consistency index also suggests that the substitution rate is very low. Thus at this level of evolution, once a point mutation occurs, there is little likelihood that subsequent mutations will occur at that site and confound the phylogenetic signal (Keim et al. 2004; Pearson et al. 2004). Finally, the high consistency index (low homoplasy) among a large number of parsimony-informative characters distributed across the genome, as well as 100% bootstrap support for each node, suggests a highly accurate phylogenetic topology.

### Assessing Outgroup Taxa for Rooting

We identified three taxa that share portions of their genomes and could therefore potentially be used to root the *C. burnetii* phylogeny. For *P. syringae*, *L. pneumophila*, and *R. grylli*, comparisons to the 10 *C. burnetii* genomes yielded only a single parsimony-informative SNP out of the 9096 parsimony-informative SNPs found among the 10 *C. burnetii* genomes. Six other parsimony-informative SNPs were found when we dropped the requirement for sites to be present in all *C. burnetii* genomes. These seven parsimony-informative SNPs result in a tree with a consistency index of 1.0 and place the root with the RSA493 and AustQD genomes (Supplementary Material S2). The lack of homoplasy is suggestive of a well-supported tree, however the reliance on only seven SNPs provides little confidence in the resulting relationships (bootstrap support values range from 59 to 71). Indeed, given the relatively vast evolutionary distance to these outgroups, multiple substitutions per site are likely and, coupled with short and unequal branch lengths, would result in incorrect placement of the outgroup.

### Using Duplicated Regions for Rooting

After filtering duplicated regions to exclude mobile elements, complex duplications, short duplications, and duplications without parsimony-informative SNPs (see “*Finding and Assessing Duplicated Regions*” in the Methods section), orthologs of the remaining five duplicated and two triplicated regions were identified (Fig. 3a–c). Orthologous regions were easily distinguished and not confused across paralogs, as orthologs are more similar to each other than they are to paralogs (Fig. 3b). Alignments of orthologs yielded 59 parsimony-informative SNP sites (Fig. 3d). For each SNP site, the polymorphic paralogs were merged and combined with a concatenation of the monomorphic paralogs assigned to a replicate set of taxa (Fig. 3d). In the resulting phylogenetic tree, the point where the two replicate groups intersect (Fig. 4a), indicates the root and either group can be used an outgroup to root the tree (Fig. 4b). The resulting maximum parsimony tree had a consistency index of 0.8551 and 100% bootstrap support for each node (Fig. 2b,c). Of the 49 non-homoplastic SNP sites, 33 placed the root on the branch leading to Q154 and Q321, with 16 on one side of the root and 14 on the other. Of the 10 homoplastic SNPs, 7 placed the root in 5 different phylogenetic positions. The best supported alternative root position was along the branch leading to Q212, albeit with only three SNPs. The topology and root position of trees inferred through Bayesian approaches were identical to the maximum parsimony tree. After determining the root position using paralogous regions, we were able to map this root position onto the more robust and better supported WGS SNP phylogeny (Fig. 4c). This tree and the 11,386 orthologous SNPs can be found in TreeBASE (http://purl.org/phylo/treebase/phylows/study/TB2:S14254).

### Biological Inferences from a Rooted Tree

*Genome reduction.—*With a well-supported root, the evolution of genetic characteristics in *C. burnetii* can be better determined. For example, genome reduction is often associated with the emergence of novel pathogens (Moran 2002; Losada et al. 2010) and may provide insights into genes that are superfluous or incompatible with virulence (Bliven and Maurelli 2012). The genome size of *C. burnetii,* coupled with the presence of pseudogenes, is suggestive of reductive evolution (Seshadri et al. 2003). The Dugway chromosome, however, is 163 kb larger than the smallest chromosome (RSA493), suggesting that genome reduction may not have occurred in all lineages or some genomes may have incorporated DNA from other species. To investigate this, we measured a pan genome size of 2148 genes and found 341 genes present in some genomes, but absent in others (Fig. 5).

*Coxiella burnetii* replicates in an exclusive intracellular niche (Howe et al. 2002) where there may be few or no opportunities to gain exogenous DNA, yet the differential presence of 116 genes among *C. burnetii* genomes is more parsimoniously ascribed to addition rather than deletion events. A majority of these genes (104/116) are found only in one genome (98 in Dugway and 6 in Q321). However, BLAST comparisons against the NCBI non-redundant database found only eight matches to other species with a fractional identity above 0.6 and none above 0.8. The lack of homologs in other species suggests that most, if not all of these genes were inherited from the last common *C. burnetii* ancestor and lost in multiple independent deletion (Fig. 5a) events rather than gained through lateral gene transfer with another species. It is important to note that while 341 genes are differentially present among *C. burnetii* genomes, many are adjacent to each other and would have been deleted from ancestral genomes in single events.

Functional characterization of the 341 *C. burnetii* accessory genes can provide insights into pathoadaptation (Bliven and Maurelli 2012), however extensive characterization is lacking with 56% being hypothetical and unknown (Fig. 5b). Many of the genes missing only from Q321 may be due to an assembly error as they are ribosomal genes thought to be essential for protein synthesis (Fig. 5a,b). Grouping the characterized genes into broad functional categories shows that many of these genes are associated with cellular processes and metabolism (Fig. 5b) and may have been required in a more diverse ancestral niche. Similarly, genes coding for enzymes and transcription/translation regulators may also be linked to metabolic functions and may be extraneous or deleterious once metabolic pathways are disrupted. However, some missing genes appear to be important in contemporary pathogen–host relationships, indicating functional redundancies. For example, ankyrin repeat domains may be involved with ensuring a stable replication niche during infections (Voth et al. 2009), LuxR—family regulators thought to control growth within a host (Minnick and Raghavan 2011) and OmpA-like transmembrane domain proteins known to be immunoreactive (Beare et al. 2008). Although the genes missing from different clades vary (Fig. 5a), there is a wide overlap in function (Fig. 5b), however known virulence and information processing genes were only missing from members of a single clade. Also, BSRs (Fig. 5a) and 3057 synonymous SNPs suggest varying degrees of polymorphisms and pseudogenization among genes found in all genomes. These patterns suggest that while broad evolutionary and adaptive patterns may be similar across clades and time, we can also expect lineage-specific fine scale adaptations.

*Evolution of virulence.—*Different virulence and pathogenicity attributes of *C. burnetii* may be an example of lineage-specific adaptivity and has been the subject of historical debate (Samuel et al. 1985; Moos and Hackstadt 1987; Stein and Raoult 1993; Thiele and Willems 1994). In humans, host genetic differences and underlying health conditions confound attempts to relate clinical observations to genetic groups. With the exception of the Dugway clade, strains belonging to the clades represented here have all been associated with disease in humans (Glazunova et al. 2005; Hornstra et al. 2011; Huijsmans et al. 2011), and different clinical manifestations have been reported among patients infected with similar strains. Also, due to the large number of potential hosts and lack of reported disease in most naturally infected animals, comparisons of virulence differences have been limited, making animal models particularly valuable. These models measure virulence of acute disease that is similar in humans, but do not model chronic disease. In these animal models, isolates from the clade consisting of Q321, Q154, and Q177, cause infections at low dose challenge in guinea pigs and mice, but show no significant pathology at low or high doses (Moos and Hackstadt 1987; Russell-Lodrigue et al. 2009). Similarly, isolates in the Dugway clade on the other side of the root, were originally reported as low or avirulent for acute disease in rodents and this was recently confirmed in mice and guinea pig models (Stoenner et al. 1959; Stoenner and Lackman 1960; Russell-Lodrigue et al. 2009). Several isolates in this group were obtained from chronic human cases or goat abortions, possibly representing a persistent (chronic) infection in goats as well as humans. Indeed, although variable, overall trends in human acute disease manifestations seem to largely follow predictions based on animal models. Since these two clades (the Q321/Q154/Q177 clade and the Dugway clade) straddle the root, we speculate that the ancestor of *C. burnetii* was more likely to cause a persistent or chronic, as opposed to acute, infection in humans, although no animal models are available to test virulence required for chronic disease. Isolates from the clade consisting of AustQD, RSA493, M44, and RSA331, however, are highly virulent and cause acute disease in mice and guinea pigs. Isolates from the clade between the Dugway and AustQD/RSA493/M44/RSA331 clades (consisting of Q212 and McMaster) caused an intermediate type of disease (Russell et al. 2009).Given the position of the root, the evolution of high acute virulence may have involved mutations before and after the bifurcation point leading to the Q212 and McMaster clade, leading to a gradual evolution of high acute virulence in the RSA493 clade and an alternate adaptive pathway involving intermediate acute virulence in the Q212 clade.

*Geographic dispersal.—*Despite knowing the root of the *C. burnetii* tree, we are still far from establishing the geographic origin of the species or even particular clades. Geographically diverse sample collections are small and rare and most genotyping methods are not amenable to comparisons across collections (but see Hornstra et al. 2011.Geographic assignment of isolates from existing collections suggest that many major clades contain isolates from different continents (Glazunova et al. 2005; Hornstra et al. 2011), hiding patterns of deep paraphyly of isolates from a particular region that could indicate geographic origins.

## DISCUSSION

Orthologous SNPs from whole genome comparisons have been previously used to infer phylogenies for bacterial pathogens. Compared to many other types of polymorphic sites, SNPs are more evolutionarily stable and are therefore less prone to the confounding effects of homoplasy (Keim et al. 2004; Pearson et al. 2004). Here, we also use 11,386 orthologous whole genome SNPs to infer the unrooted phylogeny of *C. burnetii* that could not be rooted using the traditional outgroup rooting method. We then illustrate how extracting 59 SNPs from duplicated genomic regions (paralogs) can be used to root the recent radiation of *C. burnetii*. Previous phylogenetic analyses of *C. burnetii* used a midpoint rooting method to root the tree (Glazunova et al. 2005; Leroy et al. 2011), and while our unrooted phylogenetic topology is in agreement, our analyses do not support a midpoint root (For a rooted MST tree, see the Supplementary Material S3). Other analyses using outgroup species and protein comparisons found multiple possible root positions (Beare et al. 2009), one of which is consistent with our root position.

In small genomes among recent radiations, duplicated genomic regions may be particularly rare and only a small number of paralogous SNP mutations may have occurred. By searching whole genome sequence data, we were able to find multiple candidate duplicated regions containing SNPs. The use of multiple duplicated regions, as opposed to a single gene, has two advantages. Firstly, multiple regions provide the potential for additional characters and thus more robust trees. Secondly, the phylogenetic signal provided by multiple regions scattered throughout the genome will less likely be skewed by a history of lateral gene transfer. Using regions duplicated more than once (e.g. triplicated) may yield novel SNPs, increasing the total number of sites. Such regions will increase confidence in the root position, as phylogenetic signals produced by each paralog should be congruent. Also, by treating each SNP as a paralogous unit, rather than an entire region, all polymorphic sites can be combined to concentrate all the phylogenetic resolution into a single paralog clade. Although more sites will certainly yield more robust trees, a relatively small number of SNPs may suffice for rooting, provided a low substitution rate and resolution surrounding the root.

The age of duplicated regions appropriate for rooting may be significantly older than the most recent common ancestor. The deeper evolutionary origin of these duplications and possible lack of selective pressures to maintain the integrity of duplicated regions may explain the increased homoplasy among paralogous SNPs compared to orthologous SNPs among the ingroup. A maximum parsimony approach is valuable for inferring phylogenies of recent radiations, however for more divergent groups, other methods may be more appropriate. For our rooting analysis however, Bayesian analyses inferred the same root position.

For genetically diverse groups, whole genome comparisons can lead to the identification of paralogous regions and paralogous SNPs throughout the genome, providing more characters and thus increase phylogenetic resolution and confidence. Although identifying paralogous regions may be possible within very diverse groups, precise alignment of paralogs to correctly identify paralogous SNPs may not be possible, requiring that individual regions, rather than SNPs be used as the parolog unit. Although not all rooting methods are best suited for all phylogenetic problems (Simmons 2008), using paralogous genes in lieu of outgroups can serve as a vital alternative tool for deciphering phylogenetic roots when traditional methods fail.

With a rooted tree, the directionality of evolution can be determined and the genetic differences among taxa can provide insights into the ecology and evolution of the species. For many pathogens, addition of exogenous DNA can impart virulence and antibiotic resistance differences and result in adaptive radiations to the detriment of host species. For *C. burnetii,* this mechanism of adaptation does not appear to have happened since the radiation of the species. Rather, adaptation within this species appears to be based on modification and loss of existing gene content. Lost genes may have been superfluous, incompatible with a new environment, or inhibit virulence. Studying such genes may lead to the discovery of new therapies and vaccines. Without the ability to gain exogenous DNA, adaptation may be slower, having implications on the longevity and spectrum of such therapies for Q fever patients.

## CONCLUSIONS

Accurate rooting is essential for inferring the directionality of evolution, which increases the power of interpreting genetic changes and geographic dispersal. Using duplicated genes as proxies for outgroups provides a valuable alternative methodology for rooting when outgroups are unavailable, unknown, or provide insufficient phylogenetic information. Duplicated genes have been used to root deeply diverged groups of taxa, but as sequencing technologies increasingly facilitate genomic comparisons of groups with little genotypic variation, such regions offer an important alternative for rooting rapid radiations as well. Combining data from multiple duplicated regions, as well as across paralogs, exploits all the rare phylogenetic information that can be used to root such groups when outgroups fail.

## SUPPLEMENTARY MATERIAL

Supplementary material, including data files and/or online-only appendices, can be found at http://datadryad.org/resource/doi:10.5061/dryad.3g615 and in the Dryad data repository (doi: 10.5061/dryad.3g615).
